# Triple X syndrome in a patient with partial lipodystrophy discovered using a high-density oligonucleotide microarray: a case report

**DOI:** 10.4076/1752-1947-3-8867

**Published:** 2009-08-12

**Authors:** Matthew B Lanktree, I George Fantus, Robert A Hegele

**Affiliations:** 1 Blackburn Cardiovascular Genetics Laboratory, Room 4-07, Robarts Research Institute, University of Western Ontario, London, Ontario, N6A 5K8, Canada; 2 Mount Sinai Hospital, Lebovic Building, Rm 5-028, University of Toronto, Toronto, Ontario, M5T 3L9, Canada

## Abstract

**Introduction:**

Patients with lipodystrophy experience selective or generalized atrophy of adipose tissue. The disruption of lipid metabolism results in an increased risk for development of metabolic syndrome and coronary artery disease. Currently, the mutations responsible for approximately half of lipodystrophy patients are known, but new techniques and examination of different types of genetic variation may identify new disease-causing mechanisms.

**Case presentation:**

A 53-year-old woman of African descent was referred to a tertiary care endocrinology clinic for treatment of severe insulin resistance, treatment-resistant hypertension and dyslipidemia. After all known lipodystrophy-causing mutations were excluded by DNA sequencing, the patient was found to have triple X syndrome after an initial investigation into copy number variation using a high-density oligonucleotide microarray. The patient also had a previously unobserved duplication of 415 kilobases of chromosome 5q33.2. This is the first case report of a patient with lipodystrophy who also had triple X syndrome.

**Conclusion:**

While we cannot make a direct link between the presence of triple X syndrome and partial lipodystrophy, if unrelated, this is an extremely rare convergence of syndromes. This patient poses an interesting possibility regarding the influence triple X syndrome may have on an individual with other underlying lipodystrophy susceptibility. Finally, impending large-scale case-control and cohort copy number variation investigations will, as a by-product, further document the prevalence of triple X syndrome in various patient groups.

## Introduction

Lipodystrophies are a family of disorders characterized by selective or generalized atrophy of adipose tissue [1]. The molecular mechanisms of many forms of lipodystrophy have been discovered by carefully selecting patients to ensure phenotypic homogeneity and performing molecular genetic analysis to identify mutations. Sequencing of both functional candidate genes, from knowledge of lipid metabolism pathways and model organisms, and positional candidate genes, identified through linkage analysis, have uncovered mutations in genes responsible for two subtypes of congenital generalized lipodystrophy (*AGPAT2, BSCL2*), three subtypes of familial partial lipodystrophy *(LMNA, PPARG, CAV1*), and some patients with acquired partial lipodystrophy (*LMNB2*) [2]. The metabolic syndrome, a constellation of symptoms including dyslipidemia, specifically increased plasma triglycerides and decreased high-density lipoprotein cholesterol (HDL), dysglycemia, insulin resistance, increased visceral obesity and increased cardiovascular risk, is common in patients with lipodystrophy [2]. Lipodystrophy mutations may have a direct impact on pathways of insulin resistance and lipid metabolism; thus, the identification of additional molecular mechanisms could improve insight into the more common form of metabolic syndrome. Currently, ~50% of patients with clinically diagnosed lipodystrophy have no known molecular basis (RAH, unpublished observations). The development of new strategies and the examination of new types of variation are required to further elucidate lipodystrophy pathogenesis.

Copy number variations (CNVs) were first identified as a relatively high frequency source of genetic variation in 2004 [3]. CNVs are submicroscopic deletions or duplications of genomic DNA above the resolution of sequencing techniques (>~1 kb). Since 2006, large efforts have been made to categorize and map the spectrum of CNVs in the unaffected population [4]. Here we report a patient with partial lipodystrophy who had no mutations in any known lipodystrophy gene and who we subsequently found to have triple X syndrome during genome-wide screening to detect lipodystrophy-associated CNVs using oligonucleotide microarrays.

## Case presentation

A 53-year-old woman from Ghana was referred for severe insulin resistance, treatment-resistant hypertension and dyslipidemia. She was first diagnosed with type 2 diabetes at age 40 and was treated with oral hypoglycemic agents until age 51 when insulin was added. She presented to the emergency room with exertional chest heaviness, dyspnea, and epigastric discomfort, however no definite cardiac event was diagnosed. She had a history of burning pain and numbness in her feet, diffuse muscle pains consistent with a chronic pain syndrome, and bilateral frozen shoulders. The patient has a strong family history for early coronary heart disease: her father had a myocardial infarction (MI) in his early 60s, her mother had three MIs, one sister had coronary artery bypass surgery in her 40s and another sister had an MI in her 30s. The patient's only family history of diabetes was in her maternal grandfather's brother.

Current medications included 120 units of insulin daily, amlodipine, valsartan, hydrochlorothiazide, rosuvastatin, ezetimibe, metformin, enteric coated acetylsalicylic acid (ASA), rabeprazole, amitriptyline, meloxicam, K-lyte, and magnesium.

On physical examination, she had a typical Dunnigan-type lipodystrophic habitus, including lipoatrophy of the lower extremities but sparing of fat deposits in the abdominal region, face and neck. Although the age of onset of her lipodystrophy is not known, she has had the same physiognomy for most of her adult life. Her radial pulse was 70 beats/minute and blood pressure was 150/85 mmHg. Her weight was 61.8 kg, height 162 cm, and body mass index 23.5 kg/m^2^. On cardiovascular examination, soft bruits were heard over both carotids, a 2/6 systolic ejection murmur was heard over the pericardial region and transient pitting edema of the lower extremities was detected bilaterally. On neurological examination, she had absent ankle jerk reflexes and decreased sensation in the area of the upper shin. She had marked acanthosis nigricans on the back of her neck. No other pertinent findings were observed on physical examination; specifically no ocular, dermatological, or abdominal findings.

Her laboratory results included (reference range) fasting glucose of 8.8 mmol/L (4.0-6.0 mmol/L), glycated hemoglobin 16.9% (4.1-6.5%), total cholesterol 7.2 mmol/L (4.3-6.5 mmol/L), triglycerides 3.35 mmol/L (0.67-3.15 mmol/L), low-density lipoprotein cholesterol (LDL) 4.8 mmol/L (2.3-4.3 mmol/L), HDL 0.9 mmol/L (1.0-2.4 mmol/L). Cardiac investigations, including electrocardiogram, echocardiogram, exercise and pharmacological stress echocardiogram, gated single photon emission computed tomography (SPECT) perfusion study, cardiac catheterization and coronary angiography, were all negative specifically showing no significant ischemia or coronary artery disease.

**Figure 1:**
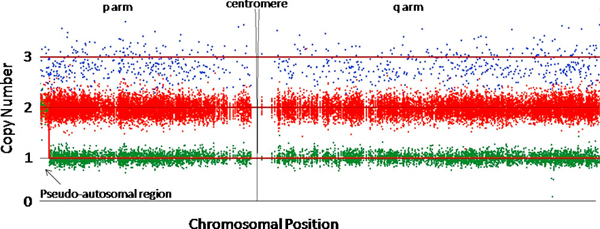
**X chromosome probe intensity for copy number determination**. Copy number determination was performed using 130 normal controls (65 men, 65 women) and 40 lipodystrophy cases (29 women, 11 men). Red dots represent single nucleotide polymorphism intensity for 29 female lipodystrophy patients. Green dots represent SNP intensity for 11 male lipodystrophy patients. Blue dots represent single nucleotide polymorphism intensity for the female lipodystrophy patient diagnosed with triple X syndrome. For the patient with triple X syndrome, probe intensities were within the normal range for all other chromosomes, with the exception of the duplication on 5q33.2 (data not shown).

The patient was found to carry no mutation in any of the known lipodystrophy-causing genes (*LMNA, PPARG, BSCL, AGPAT2, LMNB2, CAV1*) by exon-by-exon sequence analysis. Genomic DNA was extracted from peripheral blood leukocytes and 250 ng was used in each genotyping assay. The high-density oligonucleotide microarrays (chips) used for analysis were the Affymetrix GeneChip Human Mapping 500 K array set (Santa Clara, CA, USA). The chips were processed using established protocols in the London Regional Genomics Centre. Copy number determination was performed using 130 normal controls and 40 lipodystrophy cases (Figure 1) with Partek Genomics Suite version 6.3 (St Louis, MO, USA). No log transformations or data normalizations were used, but probes were corrected for fragment length, sequence, and guanine-cytosine (GC) content. Two CNVs were identified within the genome of the patient: three copies of the entire X chromosome, and a duplication of 89 single nucleotide polymorphism (SNP) probes over 415 kb of chromosome 5q33.2, from 154,979 kb to 155,394 kb (physical position, NCBI reference build 36.2). No genes were found in or within 300 kb of the 5q33.2 duplication. Neither the duplication of 5q33.2, nor triple X syndrome, was identified in any of the other lipodystrophy patients or normal controls.

## Discussion

We report the case of a patient with partial lipodystrophy, who had no known molecular diagnosis, and who was discovered to be a carrier of three X chromosomes after genome-wide screening for CNVs (Figure 1). To our knowledge, this is the first report of a patient with both triple X syndrome and partial lipodystrophy. An additional 40 lipodystrophy patients with no known mutation were investigated using a high-density oligonucleotide microarray: among the 29 female lipodystrophy patients, no other cases of triple X syndrome were observed. A comprehensive amniocentesis screen of 34,910 children for sex chromosome abnormalities reported the prevalence of triple X syndrome as 1 in 947 girls [5]. Thus, the observation of a lipodystrophy patient with triple X syndrome in a cohort of 29 women was surprising. Partial lipodystrophy is rare, but estimates of prevalence are difficult due to the heterogeneity of clinical diagnosis [6]. While collection of lipodystrophy patients is difficult due to the low disease prevalence, karyotyping of additional lipodystrophy patients would give better insight into the frequency of triple X syndrome in this patient cohort. We cannot make a direct link between triple X syndrome and the lipodystrophy found in this patient, however it does pose an interesting possibility regarding the influence triple X syndrome may have on an individual with other underlying susceptibility. If the triple X syndrome and lipodystrophy found in this patient are incidental and unrelated, this convergence of syndromes would be extremely rare.

The CNV screen identified a large 415 kb duplication of chromosome 5q33.2. To our knowledge, this duplication has not been previously identified in a lipodystrophy patient, in our normal controls, or in the public database of genomic variants [3]. A large degree of CNV in this size range has been identified in the 'healthy' population [3]. There are no known genes in or within 300 kb of the duplication and no previously identified lipodystrophy loci are found on chromosome 5q. Therefore, we conclude that there is insufficient evidence at this point to support further investigations specifically into a potential function for the 5q33.2 duplication.

The most commonly reported findings in triple X syndrome are tall stature, mild mental retardation, behavioral problems, and premature ovarian failure [7]. None of the common findings in triple X syndrome were present in our patient. Because karyotype analysis is not part of the routine work-up for a patient with partial lipodystrophy, our study is an example of how a large cytogenetic abnormality that would otherwise remain undiagnosed can be serendipitously identified after CNV screening using high-density oligonucleotide arrays. Attempts to map structural changes in healthy control populations have revealed a large degree of variation [4]. As studies screen large case-control and cohort datasets for CNV changes we will, as a by-product, further refine the frequency and impact of large structural variations.

Within the literature, triple X syndrome cases have been reported with gastrointestinal, renal, and urogenital abnormalities [8], as well as gonadal dysgenesis, congenital adrenal hyperplasia, and central precocious puberty [9]. Two triple X syndrome patients have been reported with insulin resistance. One teenaged patient mosaic for triple X syndrome had insulin resistance, tall stature and disturbed behavior [10]. A second patient had transient neonatal diabetes mellitus and later type 2 diabetes diagnosed at age 31, but was found to have a concomitant uniparental paternal isodisomy of chromosome 6, subsequently linked to type 2 diabetes [11].

One limitation of the use of oligonucleotide arrays to determine copy number is that it is not possible to determine whether all cells contain the same genomic structure. Patients with triple X syndrome have been reported to be mosaic for chromosome number, such as 46, XX/47, XXX [10]. Moreover, without parental genotype information, we are unable to determine the parental source of the additional X chromosome.

In women, X-linked gene dosage equivalency is created by the X-inactivation (also known as lyonization) of either the maternally- or paternally-inherited X chromosome, as well as any extra X chromosomes, in the late blastocyst stage [12]. The inactive state is retained in all cells through epigenetic inheritance [12]. Thus, men and women have one transcriptionally active copy of the X chromosome. Pseudo-autosomal regions of X, found near the telomere of the short arm, are excluded from X-inactivation and are also found on the Y chromosome, so all humans with two sex chromosomes have two transcriptionally active copies of these regions [12]. Genes that escape X-inactivation are excellent candidates for involvement in the triple X phenotype. The *SHOX* gene lies within the pseudo-autosomal region and overdosage, as a result of triple X syndrome, when combined with estrogen deficiency, has been suggested to be responsible for the often tall stature in women with three X chromosomes [10].

Insulin resistance and other metabolic disturbances are more commonly identified in men with an extra X chromosome [13]. The mechanism of insulin resistance in patients with Klinefelter syndrome is unknown, but has been suggested to be primarily mediated by a decrease in insulin sensitivity due to increased truncal obesity and decreased muscle mass secondary to hypogonadism [13]. However, testosterone treatment only partly corrected the metabolic problems of a sample of patients [13]. Perhaps an unknown factor on the additional X chromosome is involved in the unfavorable fat distribution and insulin resistance.

## Conclusion

We report a patient with partial lipodystrophy including severe insulin resistance in the absence of any known lipodystrophy causing mutations discovered to have triplication of the X chromosome during genome-wide screening for CNV using a high-density oligonucleotide microarray. This is the first report of a patient with both lipodystrophy and triple X syndrome. As studies searching for genetic determinants of various disorders screen large patient populations for CNVs, we will gain greater insight into not only the effect of small structural variations, but as a by-product, the frequency and impact of triple X syndrome.

## Abbreviations

*AGPAT2*: 1-acylglycerol-3-phosphate O-acyltransferase 2 gene; ASA: acetylsalicylic acid; *BSCL*: Berardinelli-Seip congenital lipodystrophy gene; *CAV1*: caveolin 1 gene; CNV: copy number variation; GC: guanine-cytosine; HDL: high-density lipoprotein cholesterol; LDL: low-density lipoprotein cholesterol; *LMNA*: nuclear lamin A gene; *LMNB2*: nuclear lamin B2 gene; MI: myocardial infarction; NCBI: National Center for Biotechnology Information; *PPARG*: peroxisome proliferator-activated receptor gamma gene; *SHOX*: short stature homeobox gene; SNP: single nucleotide polymorphism; SPECT: single photon emission computed tomography.

## Consent

Written informed consent was obtained from the patient for publication of this case report and any accompanying images. A copy of the written consent is available for review by the Editor-in-Chief of this journal.

## Competing interest

The authors declare that they have no competing interests.

## Authors' contributions

IGF identified the patient and referred her to RAH for genetic analysis. ML analyzed and interpreted the microarray results and drafted the manuscript. All authors participated in manuscript production and approved the final version.
